# Factors predicting long-term outcomes following physiotherapy in patients with subacromial pain syndrome: a secondary analysis

**DOI:** 10.1186/s12891-024-07686-6

**Published:** 2024-07-24

**Authors:** Thilo Oliver Kromer, Matthias Kohl, Caroline H.G. Bastiaenen

**Affiliations:** 1 Faculty of Health, Safety, Society, Furtwangen University, Study Center Freiburg, Konrad-Goldmann-Straße 7, 79100 Freiburg, Germany; 2 Caphri Research Institute, Research line Functioning & Rehabilitation, Maastricht University, Maastricht, The Netherlands; 3 Faculty of Medical and Life Sciences, Institute of Precision Medicine, Campus Villingen- Schwenningen, Furtwangen University, Furtwangen, Germany; 4 Research line Functioning & Rehabilitation, Maastricht University, Maastricht, The Netherlands; 5 Department of Epidemiology, Maastricht University, Maastricht, The Netherlands

**Keywords:** Shoulder impingement syndrome, Physical therapy modalities, Rehabilitation, Clinical decision rules

## Abstract

**Background:**

Although patients with shoulder complaints are frequently referred to physiotherapy, putative predictive factors for outcomes are still unclear. In this regard, only a limited amount of scientific data for patients with subacromial pain syndrome exist, with inconsistent results. An improved knowledge about the ability of baseline variables to predict outcomes could help patients make informed treatment decisions, prevent them from receiving ineffective treatments, and minimize the risk of developing chronic pain.

**Aim:**

The aims of this secondary longitudinal analysis are threefold: First, to investigate baseline differences between patients with and without successful long-term outcomes following physiotherapy. Second, to compare the predictive ability of two sets of putative predictive variables on outcomes, one based on the literature and one based on the data of the original trial. Third, to explore the contribution of short-term follow-up data to predictive models.

**Methods:**

Differences between responders and nonresponders were calculated. The predictive ability of variables defined through literature and of variables based on the Akaike Information Criterion (AIC) from the original trial dataset on the Shoulder Pain and Disability Index and the Patients’ Global Impression of Change at the one-year follow-up were analyzed. To test the robustness of the results, different statistical models were used. To investigate the contribution of follow-up data to prediction, short-term data were included in the analyses.

**Results:**

A sample of 87 patients with subacromial pain syndrome was analyzed. 77% (*n* = 67) of these participants were classified as responders. Higher expectations and short-term change scores were positive, and higher fear avoidance beliefs, greater baseline disability and pain levels were negative predictors of long-term outcomes in patients with subacromial pain syndrome.

**Conclusions:**

Although our results are in line with previous research and support the use of clinical factors for prediction, our findings suggest that psychological factors, especially patient expectations and fear avoidance beliefs, also contribute to long-term outcomes and should therefore be considered in the clinical context and further research. However, the hypotheses and recommendations generated from our results need to be confirmed in further studies due to their explorative nature.

**Trial registration:**

The original trial was registered at Current Controlled Trials under the trial registration number ISRCTN86900354 on March 17, 2010.

**Supplementary Information:**

The online version contains supplementary material available at 10.1186/s12891-024-07686-6.

## Introduction

Shoulder pain is a common musculoskeletal complaint in primary care [1]. Prevalence data for the general population vary considerably, ranging from 6.9 to 26%, for point prevalence and from 4.7 to 46.7% for 1-year prevalence respectively [2]. For Germany, the 1-year prevalence is approximately 4.5%, with a hospitalization rate of 3.1% [3]. Patients with shoulder pain are frequently referred to physiotherapy as initial treatment [4]. However, up to 76% of patients still have complaints 12 months after initial onset [5]. About 75% of patients presenting to primary care with shoulder pain present signs of subacromial pain syndrome (SPS) [6].

Although evidence indicates that some treatment modalities, such as exercise, are more effective than others [7], differences are often marginal or disappear over time [8, 9]; moreover, surgery has comparable long-term effects to those of physiotherapy [10]. Possible explanatory hypotheses for this phenomenon are that the natural course of this pathology might be more influential than the impact of any intervention applied [11] or that long-term outcomes are more influenced by factors other than the intervention itself; thus, the results are comparable to those of a wait-and-see strategy or sham treatment [10, 12]. Research has identified a variety of potential contributing factors to shoulder pain, such as rotator cuff function [13], shoulder girdle stability [14], and posture [15]. However, it is still unclear which factors are related to a relevant outcome following physiotherapy for shoulder pain. In addition to body structures, clinical baseline characteristics such as pain intensity, duration of complaints and disability seem to have a substantial influence on both subacute and chronic complaints [16–18]. In a large cohort study by Chester et al. [19], better patient-rated outcomes following physiotherapy were associated with lower baseline disability, greater patient expectations, greater pain self-efficacy, and less severe pain at rest. For patients with SPS, only a few studies exist with inconsistent results. For example, low education, previous episodes of shoulder pain, higher disability and pain baseline scores, and psychological factors are considered putative predictors of outcomes in this subgroup [20, 21]. Since the current recommendation for an evidence-based treatment for SPS is exercise therapy, it may also be useful to monitor the development of pain and disability scores over the first few weeks and to analyze to what extent changes in these parameters contribute to prediction. Therefore, improved knowledge about the predictive capacity of baseline and short-term change factors for long-term outcomes in SPS could help patients make informed treatment decisions, prevent them from being ineffective and thus unnecessary, and minimize the risk for the development of chronic pain. It could also help care providers decide whether to continue physiotherapy, refer patients to multidisciplinary treatment, check indications for surgery, or initiate a more extensive diagnostic process. Thus, the aims of this analysis are threefold:

Aim 1: To investigate baseline differences between patients with and without a successful long-term outcome, measured with the Patients’ Global Impression of Change scale (PGIC), for all data measured at baseline following physiotherapy.

Aim 2: To explore and compare the predictive ability of two sets of baseline variables regarding their contribution to the Shoulder Pain and Disability Index and Patients’ Global Impression of Change one year after commencing treatment (SPADI-1Y, PGIC-1Y).

Aim 3: To explore the contribution of pain and disability change scores at 5 weeks to the prediction of SPADI-C1Y and PGIC-1Y scores, these data were included in the analyses.

## Materials and methods

### Study population

Data analyzed were gathered from participants with SPS who participated in a randomized controlled trial investigating the effect of manual therapy and exercise in this patient group in Germany [9, 22]. Ethical approval for the original trial was granted by the ethics committee of the Ludwig-Maximilians-University Munich, Germany (project no. 018 − 10). The trial was registered at Current Controlled Trials on March 17, 2010 (ISRCTN86900354). Patients were included in this trial if they were (1) between 18 and 75 years old, (2) had symptoms for at least four weeks, presented with clinical signs of SPS, and provided informed consent for participation and publication of the data. The study design the inclusion process, and a detailed description of the interventions have been described previously [22, 23].

### Data

The data used for this analysis are displayed in Table 1. A brief description of the most important measurements is provided below, and a more comprehensive description can be found in the protocol of the original paper [23].

**Table 1:** Baseline demographic and clinical data, differences between responder and nonresponder

	Total group (*n* = 87)			Responder (*n* = 67)			Nonresponder (*n* = 20)			Between group differences (*p*-values)		
	Median (IQR)	% / Mean (SD)	*n*	Median (IQR)	% / Mean (SD)	*n*	Median (IQR)	% / Mean (SD)	*n*	Welch two sample t-test	Wicoxon rank sum test	Fisher’s Exact Test
Age (years)	51.0 (18)	52.0 (11.4)		51.0 (18)	52.3 (11.5)		51.5 (15.8)	50.1 (11.0)		0.62	0.61	
Gender (female)		49.4	43		52.2	35		40.0	8			0.45
BMI	25 (4.5)	25.9 (4.1)		25.2 (4.3)	26 (4)		24.4 (3.1)	25.8 (4.5)		0.85	0.44	
PWL (high)		13.8	12		13.4	9		15	3			0.86
DSL (yes)		5.7	5		6.0	4		5.0	1			0.74
DSL	0 (0)			0 (0)			0 (0)				0.85	
DOC (weeks)	36 (92)			32 (88)			100 (182)				0.15	
DOC > 36 weeks		48.3	42		43.3	29		65.0	13			0.13
PE (yes)		33.3	29		32.8	22		35.0	7			1.0
PI	5 (2)	5.1 (1.8)		5 (2)	4.9 (1.6)		6 (3.2)	5.8 (1.9)		0.09	0.10	
SPADI	38.3 (25)	41.0 (17.0)		37.3 (19.5)	38.2 (16.7)		51.6 (25)	50.4 (15.1)		0.004*	0.003*	
SPADI > 38 (yes)		48.3	42		43.3	29		65	13			0.13
SPADI-P	45.8 (27)	49.3 (17.9)		43 (24.3)	45.9 (17.4)		61.8 (22.5)	60.6 (15.3)		0.001*	0.001*	
SPADI-*P* > 46 (yes)		47.1	41		37.3	25		80.0	16			0.001*
SPADI-F	29.6 (30)	32.7 (18.9)		27.8 (27)	30.5 (18.6)		36.4 (30.4)	40.1 (18.5)		0.05*	0.042*	
SPADI-F > 30 (yes)		47.1	41		44.8	30		55.0	11			0.45
FABQ-PA	16 (7)	14.5 (4.9)		15 (6)	14.0 (4.7)		18 (7.5)	16.4 (5.0)		0.06	0.04*	
FABQ-W	11 (16)	11.6 (9.7)		10 (16)	10.9 (9.1)		13 (14.8)	13.9 (11.5)		0.31	0.42	
PCS	9 (11)	11.4 (8.6)		9 (10)	10.3 (7.1)		13.5 (20.5)	15.0 (11.9)		0.11	0.16	
PET	9 (2)	8.4 (1.6)		9 (2)	8.9 (1.1)		8 (1)	7.7 (2.2)		0.036*	0.02*	
PET ≥ 9 NRS (yes)		56.3	49		64.2	43		30.0	6			0.01*
REL (yes)		31.0	27		28.4	19		40.0	8			0.41
RER (yes)		26.4	23		20.9	14		45.0	9			0.03*
CCx (yes)		55.2	48		53.7	36		60	12			0.79
PI-C	2 (3)	2.0 (2.1)		2 (2)	2.0 (2.0)		1 (4)	1.9 (2.5)		0.79	0.51	
SPADI-C	13.9 (22)	15.8 (17.6)		15.2 (19)	16.7 (18.1)		7.2 (28.6)	12,6 (16.1)		0.341	0.333	
SPADI-PC	17.8 (27)	19.5 (21.8)		19.2 (26.3)	19.6 (20.9)		10.8 (31.8)	19.1 (25.5)		0.92	0.61	
SPADI-FC	11.9 (21)	13.1 (17.3)		13.5 (18.7)	13.9 (18.1)		6.1 (21)	10.5 (14.5)		0.38	0.431	

IQR = Interquartile range; SD = Standard deviation; BMI = Body mass index; PWL = Physical work load; DSL = Days of sick leave at baseline; DOC = Duration of complaints; PE = Previous episodes last 12 months; PI = Average pain intensity on numeric rating scale; SPADI = Shoulder Pain and Disability Index; SPADI-*P* = Shoulder Pain and Disability Index-pain subscale; SPADI-F = Shoulder Pain and Disability Index-function subscale; FABQ-PA = Fear Avoidance Beliefs Questionnaire-physical activity subscale; FABQ-W = Fear Avoidance Beliefs Questionnaire-work subscale; PCS = Pain Catastrophizing scale; PET = Patients’ expectancies about treatment; REL = Restriction of passive elevation; RER = Restriction of passive external rotation; CCx = Comparable signs of the cervical spine; PI-C = Pain intensity-change score at 5 weeks; SPADI-C = SPADI change score at 5 weeks; SPADI-PC = SPADI-P change score at 5 weeks; SPADI-FC = SPADI-F change score at 5 weeks; *=*p* < 0.05

### Outcome measures (dependent variables)

The SPADI-1Y and the PGIC-1Y were used as the dependent variables for this analysis.

The SPADI is a valid and highly responsive shoulder-specific questionnaire that is often used as a primary outcome measure in studies about shoulder pain. It contains 5 items assessing pain and 8 items assessing shoulder function. The total score ranges from 0 to 100, with higher scores reflecting higher pain/disability levels. The German version of the SPADI also shows excellent reliability and internal consistency for both the total score (SPADI) and its subscores for pain (SPADI-P) and function (SPADI-F) [24]. The PGIC captures the patients’ subjectively perceived overall change in complaints since the commencement of the intervention and is therefore an important indicator of its success. Patients rated their improvement on a 5-point Likert scale from “much worse” to “much better” 52 weeks after inclusion in the study. For the analysis of the PGIC-1Y, the scale was dichotomized; a rating of “much better” was defined as a successful outcome (responder); all other ratings were defined as an unsuccessful outcome (nonresponder).

### Putative predictors

All baseline data displayed in Table 1 were considered potentially predictive for outcomes, including baseline data for the total SPADI score, its subscales for pain (SPADI-P) and function (SPADI-F).

Pain catastrophizing was measured with the Pain Catastrophizing Scale (PCS), a multidimensional, reliable, and valid 13-item measurement tool strongly associated with pain and emotional distress [25]. The PCS has been validated for the German population [26] and comprises three subscales for rumination, magnification, and helplessness. Each item is rated on a 5-point scale from 0 (not at all) to 4 (all the time). The total score and subscores for each subscale are calculated by summing the ratings for each item within a subscale.

Fear avoidance beliefs were assessed with the Fear Avoidance Beliefs Questionnaire (FABQ), a common and valid 16-item questionnaire for measuring fear avoidance beliefs in patients with low back pain. The German version shows good psychometric properties [27]. Each item is scored on a seven-point Likert scale (0 = strongly disagree, 6 = strongly agree). The total score was calculated by summing the resultant scores, with higher scores reflecting stronger fear avoidance beliefs. It comprises two subscales, one for physical activity (FABQ-PA) and one for work activities (FABQ-W). To adapt the FABQ to our patient group, the word “back” was replaced by the word “shoulder”. This modification has been used to investigate anatomic areas other than the lower back [28]. Pain intensity (PI) was defined as the average weekly pain score, measured with an eleven-point numeric rating scale (NRS), duration of complaints (DOC) was measured in weeks, and previous episodes during the last 12 months (PE) were counted as numbers.

Patients’ expectancies about the success of the applied treatment (PET) were measured with a modified question of the Credibility/Expectancy Questionnaire (CEQ), developed by [29], which shows high internal consistency and good test-retest reliability. The question is “By the end of the therapy period, how much improvement in your limitations due to shoulder pain do you think will occur? The questions are scored on an 11-point NRS ranging from 0 (no improvement) to 10 (completely recovered). Higher scores reflect expectancies that are more positive. For the analysis, the scale was recoded with 10 meaning “no improvement” and 0 meaning “completely recovered”.

Hypothesizing that a prediction for an outcome one year in advance becomes more precise when factors reflecting initial development under treatment are included [30], we also included the 5-week change scores of the SPADI (SPADI-C) and its subscales for pain and function (SPADI-PC, SPADI-FC) and the PI change score (PI-C) in the analyses for both outcomes.

### Statistical analysis

#### Study population

The data were quality checked, and descriptive statistics were generated for the total group.

The baseline differences between responders and nonresponders were calculated. Responders were defined as patients who rated their final assessment on the PGIC-1Y scale with “much better”. This cut-off was chosen to increase the probability that this statement reflects a meaningful improvement [31]. Responders were coded as 1, nonresponders were coded as 0, and dichotomized baseline variables were coded accordingly.

Subsequently, baseline differences were calculated between responders and nonresponders with the Welch t test, Wilcoxon rank sum test, or Fisher’s exact test, depending on the level of the data.

#### Selection of predictive variables from the literature and model calculations

For this analysis two datasets were derived from the variables available from the original trial according to different criteria. Dataset one, referred to as the literature-based dataset (LB-dataset), contained baseline variables identified as relevant predictors from two systematic reviews [17, 18] and one large prospective cohort study [19] investigating patients with shoulder pain. From these three studies, five baseline factors could be identified that were also available in our dataset, namely, age, pain and disability scores, duration of complaints, and patient expectation of recovery.

Dataset two, referred to as the trial-based dataset (TB-dataset), was identified through a stepwise model selection based on the Akaike Information Criterion (AIC).

To explore the predictive ability of the LB dataset, different statistical models were calculated and compared for the SPADI-1Y and the PGIC-1Y. For the SPADI-1Y as a continuous measure, an analysis of covariance (ANCOVA) was calculated for the total group, adjusted for the included baseline variables. Subsequently, the residuals were checked for normality and heteroscedasticity. If necessary, possible problems were addressed with the R package “bestNormalize” [32], resulting in refined models.

For the use of the PGIC-1Y as a binary outcome (responders and nonresponders), logistic regression analysis was used. The area under the curve (AUC) was calculated, and a receiver operating characteristic (ROC) curve was generated. In the case of unequal group sizes between responders and nonresponders, the standard cutoff (0.5) was adapted to actual group sizes based on the Youden J statistics to put equal weight on sensitivity and specificity. Finally, and if appropriate, the model calculation was repeated, replacing continuous independent variables with their dichotomized counterparts.

#### Selection of predictive variables from the TB dataset and model calculations

For the SPADI-1Y, stepwise model selection based on the Akaike information criterion (AIC) was conducted, including all baseline variables and the 5-week change scores of the SPADI (SPADI-C), its subscales for pain and function (SPADI-PC, SPADI-FC), and the PI change score (PI-C). As described before, possible problems were addressed, resulting in refined models. In the second step, this calculation was combined with thousand bootstrap repetitions to increase the stability of the model selection. To reduce variables in the model, if necessary, only variables that were selected in at least 60% of the bootstrap repetitions were included in the analysis. In addition, two alternative models were calculated to check the extent to which the results from the previous models could be reproduced. The first alternative model was Lasso (least absolute shrinkage and selection operator) regression [33] with the R package “glmnet” [34]; the optimal parameter lambda for the calculations was determined using 100 repetitions of a 10-fold cross-validation. The second one was a random forest regression [35] with the R package “ranger” [36].

The same models were applied for the PGIC-1Y. Additionally, the AUC was calculated, and ROC curves were generated. In the case of unequal group sizes between responders and nonresponders, the cutoff was adjusted as described before. Models calculated for each outcome were then compared to each other with the help of the package “R performance” [37]. All data analyses were performed using the statistical software package R (version 4.2.3) [38].

The application of different statistical models was based on the assumption that analysing data with different statistical models would increase the validity of the results. Different regression models vary in the way of analysing a particular data set and may therefore lead to different results, e.g. may identify different independent variables as relevant for prediction. However, we assumed that if an independent variable contributes substantially to an outcome, it should become in all or at least in most of them significant.

## Results

### Responders and nonresponders

87 of the 90 participants originally included in the original trial competed the 1-year follow-up and could be included in this secondary analysis. 77% (*n* = 67) of all participants (*n* = 87) rated their progression as “much better” and were defined as responders. 23% (*n* = 20) remained below this cutoff and were defined as nonresponders including ratings of “slightly better” (*n* = 12), “no change” (*n* = 5), and worse (*n* = 3). The descriptive statistics for the total group, responders and nonresponders are displayed in Table 1.

### Baseline differences between responders and nonresponders

Significant differences in baseline variables between responders and nonresponders were identified for the SPADI, SPADI-P, SPADI-F, FABQ-PA, PET, and restriction of shoulder external rotation (RER). These results are also shown in Table 1.

### Predictive ability of variables from the LB dataset for the SPADI-1Y and the PGIC

#### Selection of predictive variables from literature and model calculations

The forementioned five baseline factors age, pain and disability scores, duration of complaints, and patient expectation of recovery could be included in this analysis. The analysis of covariance for the SPADI-1Y, adjusted for the included baseline variables SPADI-P, SPADI-F, Age, DOC, and PET (model 1), resulted in an R^2^ (R^2^ adjusted) of 0.255 (0.209), and the AIC was 737.25. However, residuals showed clear signs of heteroscedasticity and deviation from the normal distribution. We therefore selected the square root as the simplest among the best transformations. We also transformed the baseline variable SPADI to SPADI.sqrt to adapt the scale accordingly. After recalculation (model 2), residual diagnostics no longer showed any clear abnormalities or signs of collinearity (Table 2).

**Table 2:** LB dataset, SPADI-1Y.Sqrt, model 2: analysis of Covariance (ANCOVA)

Predictors	Estimates	CI	*p*
(Intercept)	2.49	-1.75–6.68	0.248
SPADI-P.sqrt	0.26	-0.25–0.76	0.320
SPADI-F.sqrt	0.21	-0.16–0.58	0.265
Age	-0.01	-0.03–0.05	0.772
DOC	0.00	0.00–0.01	0.020*
PET	-0.40	-0.71 – -0.08	0.014*
Observations:	87		
R^2^ (R^2^ adj.):	0.259 (0.213)	AIC: 379.60	

CI = Confidence Interval; *p* = *p*-value; *=*p* < 0.05; **=*p* < 0.01; ***=*p* < 0.001; R^2^ = Coefficient of determination; R^2^ adj.=Adjusted coefficient of determination; AIC = Akaike information criterion; sqrt = square root

Model 2 was then recalculated with dichotomized data for DOC and PET because a simple linear correlation between these two variables and the endpoint could not necessarily be assumed. The residual diagnostics did not show any clear abnormalities or signs of collinearity. The results for Model 3 are displayed in Table 3. The performances of models 2 and 3 were very similar, and PET/PET ≥ 9 and DOC were identified as significant predictive variables for SPADI-1Y.sqrt.

**Table 3:** LB dataset, SPADI-1Y.Sqrt, model 3: analysis of covariance (ANCOVA, dicho)

Predictors	Estimates	CI	*p*
(Intercept)	-0.53	-3.87–2.81	0.754
SPADI-P.sqrt	0.31	-0.19–0.81	0.225
SPADI-F.sqrt	0.22	-0.15–0.58	0.240
Age	-0.01	-0.03–0.05	0.563
DOC > 36 weeks	0.41	-0.55–1.36	0.399
PET ≥ 9	-1.51	-2.46 – -0.56	0.002**
Observations:	87		
R^2^ (R^2^ adj.):	0.259 (0.214)	AIC: 379.56	

CI = Confidence Interval; *p* = *p*-value; *=*p* < 0.05; **=*p* < 0.01; ***=*p* < 0.001; R^2^ = Coefficient of determination; R^2^ adj.=Adjusted coefficient of determination; AIC = Akaike information criterion; sqrt = square root

For the PGIC-1Y, a first logistic regression model was calculated. Due to the imbalance between responders (*n* = 67 (77%)) and nonresponders (*n* = 20 (23%)), the cutoff of 0.5 resulted in a clear imbalance between sensitivity (95.5%) and specificity (40.0%). The optimal cutoff using the Youden J statistic was 0.86, which led to a sensitivity and specificity of 56.7% and 85.0%, respectively. Adjusting the cutoff to actual group sizes also led to a balanced accuracy [39, 40] of 0.86 (the accuracy for a cutoff of 0.5 was 0.68). The AUC for the model was 0.77. Overall, a rather moderate prediction accuracy was obtained by the model. Looking at the residuals, a poor model fit was observed for the nonresponders. Therefore, we replaced the baseline variables DOC and PET with their dichotomized versions. The results for this second model are displayed in Table 4. Overall, SPADI-P and PET/PET ≥ 9 were identified as significant predictive variables.

**Table 4:** LB dataset, PGIC-1Y, model 2: logistic regression, dicho

Responder			
Predictors	Odds Ratios	CI	*p*
(Intercept)	10.85	0.42–405.22	0.168
SPADI-P	0.95	0.90–0.99	0.022*
SPADI-F	1.01	0.97–1.06	0.557
Age	1.01	0.96–1.07	0.610
DOC > 36 weeks	0.95	0.27–3.40	0.937
PET ≥ 9	4.43	1.30–17.30	0.022*
Observations:	87		
R^2^ Tjur:	0.203	AIC: 88.19	

CI = Confidence Interval; *p* = *p*-value; *=*p* < 0.05; **=*p* < 0.01; ***=*p* < 0.001; R^2^ Tjur = Coefficient of determination; AIC = Akaike information criterion

Using the optimal cutoff of 0.77, the sensitivity and specificity changed from 95.5% and 20–73.1% and 75%, respectively. The balanced accuracy increased from 0.58 to 0.74. The AUC was 0.77. Model comparison resulted in a minimal advantage for Model 1, again with a poor model fit for the nonresponders. However, the AUCs were not significantly different (*p* = 0.94, test of Hanley and McNeil [41]), The generated ROC curves for the two models are shown in additional Figs. 1 and 2 (see Additional file 01).

#### Selection of predictive variables from the TB dataset and model calculations for the SPADI-1Y

Predictive variables from the TB dataset were selected by means of a stepwise model based on the AIC. For the aforementioned reasons, we again chose SPADI.sqrt as an endpoint for the first model presented in Additional Table 1 (see Additional file 02). To increase the stability of the model selection, we combined it again with a bootstrap and performed model selection with SPADI-1Y.sqrt as the response in each case. The results are shown in Additional Fig. 3 (see Additional file 03). In the second model, we included only those variables that were selected in at least 60% of the bootstrap repetitions (Table 5). The residual diagnostics showed no clear abnormalities, a good fit to normality, and only mild to moderate collinearity. Both models included a PET ≥ 9, FABQ-PA, SPADI-C and SPADI-*P* > 46 as significant variables. Model 1 showed slightly better results than did Model 2. However, the second model might be better in terms of generalizability and hence might provide better results for new data.

**Table 5:** TB dataset, SPADI-1Y.Sqrt, model 2: coefficients (bootstrap)

Predictors	Estimates	CI	*p*
(Intercept)	1.70	-0.04–3.45	0.055
PET ≥ 9	-1.42	-2.32 – -0.53	0.002**
FABQ-PA	0.10	0.00–0.19	0.042*
SPADI-C	-0.08	-0.15 – -0.02	0.012
SPADI-*P* > 46	1.78	0.85–2.70	< 0.001***
Classification of work demand: low	0.36	-0.98–1.69	0.597
Classification of work demand: med	-0.01	-1.46–1.45	0.993
Classification of work demand: high	0.10	-1.55–1.74	0.907
DSL	-0.07	-0.23–0.09	0.409
SPADI-FC	0.06	-0.00–0.12	0.065
Observations:	87		
R^2^ (R^2^ adj.):	0.377 (0.304)	AIC: 372.6	

CI = Confidence Interval; *p* = *p*-value; *=*p* < 0.05; **=*p* < 0.01; ***=*p* < 0.001; R^2^ = Coefficient of determination; R^2^ adj.=Adjusted coefficient of determination; AIC = Akaike information criterion; sqrt = square root

The first alternative model (Model 3) was calculated via Lasso (Table 6). Random forest regression (Model 4) was the second alternative. The use of this machine learning method yielded somewhat different results but also showed the predictive importance of the SPADI baseline variables and their change scores for SPADI-1Y.sqrt (see Additional file 04, Additional Fig. 4).

**Table 6:** TB dataset, SPADI-1Y.Sqrt, model 3: coefficients (model selection through Lasso)

Predictors	Estimates	CI	*p*
(Intercept)	-0.26	-2.86–2.34	0.844
PET ≥ 9	-1.09	-1.96 – -0.22	0.015*
FABQ-PA	0.10	0.01–0.19	0.026*
SPADI	0.32	-0.13–0.78	0.163
SPADI-*P* > 46	1.00	-0.21–2.22	0.104
SPADI-PC	-0.03	-0.05 – -0.01	0.009**
DOC	0.00	-0.00–0.01	0.103
DSL	-0.09	-0.24–0.06	0.229
Observations	87		
R^2^ (R^2^ adj.):	0.398 (0.345)		

CI = Confidence Interval; *p* = *p*-value; *=*p* < 0.05; **=*p* < 0.01; ***=*p* < 0.001; R^2^ = Coefficient of determination; R^2^ adj.=Adjusted coefficient of determination; AIC = Akaike information criterion; sqrt = square root

According to the TB dataset, FABQ-PA was consistently identified as a significant predictive variable in all the models, followed by PET ≥ 9 and SPADI-C in three out of the four models.

#### Selection of predictive variables from the TB dataset and model calculations for PGIC-1Y

Model selection was based on the AIC and resulted in a relatively large model 1 (see Additional file 05, Additional Table 2). The optimal cutoff (0.88) changed the sensitivity and specificity from 95.5% and 55% to 68.7% and 95%, respectively. The balanced accuracy increased from 0.75 to 0.82. The AUC for the model was 0.89. The recalculation based on bootstrapping (see Additional file 06, Additional Fig. 5) resulted in a smaller model 2, displayed in Table 7.

**Table 7:** TB dataset, PGIC-1Y, model 2: coefficients (bootstrap)

Responder			
Predictors	Odds Ratios	CI	*p*
(Intercept)	144.76	16.78–2062.38	< 0.001***
SPADI > 38	2.64	0.28–30.33	0.410
SPADI-C	1.05	1.01–1.09	0.018*
SPADI-F > 30	1.64	0.26–11.36	0.602
SPADI-P	0.91	0.85–0.96	0.002**
DOC	1.00	0.99–1.00	0.331
Observations:	87		
R^2^ Tjur:	0.242	AIC: 85.16	

CI = Confidence Interval; *p* = *p*-value; *=*p* < 0.05; **=*p* < 0.01; ***=*p* < 0.001; R^2^ Tjur = Coefficient of determination; AIC = Akaike information criterion

Here, the optimal cutoff of 0.70 changed the sensitivity and specificity from 91% and 40% to 86.6% and 75.0%, respectively, and the balanced accuracy increased from 0.66 to 0.81. The AUC was 0.82. Model comparison provided minimal advantages for the first model, but the differences were not significant (*p* = 0.266, test of Hanley and McNeil [41]). The ROC curves for models 1 and 2 are shown in Additional Figs. 6 and 7 (see Additional file 07).

An alternative third model was calculated via Lasso regression, with a sensitivity and specificity of 77.6% and 75.0%, respectively, and a balanced accuracy of 0.76. The AUC was 0.81 (see Additional file 08, Additional Table 3 and Additional Fig. 8). Finally, a random forest was calculated (model 4), as displayed in Additional Fig. 9 (see Additional file 09). This classification method provided by far the best results. Using the optimal cutoff of 0.68, both the sensitivity and specificity were 100.0%. However, we must assume that this is a so-called overfitting due to the relatively small dataset. Overall, the SPADI subscale for pain (SPADI-P, SPADI-*P* > 46) was identified in 3 models, and the SPADI-C, PET > 9, DOC, and PCS were identified in 2 out of 4 models as predictive variables for the PGIC-1Y.

Summarizing the analyses of both datasets for the SPADI-1Y.sqrt, the only variable constantly relevant for prediction was patients’ expectations. For the TB dataset, FABQ-PA was the only variable constantly identified in all models. The SPADI was not relevant in the smaller LB dataset; in the TB dataset, the SPADI-C was more important. Summarizing the analyses for PGIC-1Y, PET or PET ≥ 9 was relevant in both datasets. The SPADI-P was the second most significant predictive factor for PGIC-1Y in the smaller LB dataset, and in the TB dataset, the SPADI-C became important. An overview of these results is given in Tables 8 and 9.

**Table 8:** Overview of significant (*p* < 0.05) predictive variables for the SPADI-1Y.Sqrt identified in different models with estimates (Beta-values) and 95% confidence intervals (CI95%)

Dataset Model (description)	*R*^2^/ *R*^2^ adj.	AIC	SPADI	SPADI-*P*	SPADI-*P* > 46	SPADI-F	SPADI-C	SPADI-PC	SPADI-FC	DOC	PET	PET ≥ 9	FABQ-PA	PCS	Age
LB dataset															
Model 2 (ANCOVA)	0.259 / 0.213	379.6								0.00 (0.00–0.01)	-0.04 (0.71 – -0.08)				
Model 3 (ANCOVA, dicho)	0.259 / 0.214	379.6										-1.51 (-2.46 – -0.56)			
TB dataset															
Model 1 (AIC)	0.396 / 0.351	363.8			1.54 (0.62–2.46)		-0.09 (-0.14 – -0.03)		0.07 (0.01–0.12)	0.00 (0.00–0.01)		-1.18 (-2.03 – -0.32)	0.09 (0.00–0.17)		
Model 2 (Bootstrap)	0.377 / 0.304	372.6			1.78 (0.85–2.70)		-0.08 (-0.15 – -0.02)					-1.42 (-2.32 – -0.53)	0.10 (0.00–0.19)		
Model 3 (Lasso)	0.398 / 0.345	365.5						-0.03 (-0.05 – -0.01)				-1.09 (-1.96 – -0.22)	0.10 (0.01–0.19)		
Model 4 (Random forest^$^)			1	2		4	6	8	9	3			5	7	10

sqrt = square root; R^2^ = Coefficient of determination; R2 adj = adjusted coefficient of determination; $=first 10 most important variables ranked according to their importance; AIC = Akaike Information Criterion; SPADI = Shoulder Pain and Disability Index; SPADI-*P* = SPADI Pain subscale; SPADI-F = SPADI Function subscale; SPADI-C = SPADI change score at 5 weeks; SPADI-PC = SPADI Pain change sore at 5 weeks; SPADI-FC = SPADI Function change sore at 5 weeks; DOC = Duration of complaints; PET = Patient expectations of treatment; FABQ-PA = Fear avoidance beliefs questionnaire physical activity subscale; PCS = Pain catastrophizing scale; ANCOVA = Analysis of covariance; LB dataset = dataset based on the literature; TB dataset = dataset from the original trial

**Table 9:** Overview of significant (*p* < 0.05) predictive variables identified for the PGIC-1Y in different models with odds Ratios (OR) and 95% confidence intervals (CI95%)

Dataset Model (description)	Tjur’s *R*^2^	AIC	SPADI	SPADI > 38	SPADI-*P*	SPADI-*P* > 46	SPADI-F	SPADI-C	SPADI-PC	DOC	PET	PET > 9	FABQ -PA	PCS	BMI	PI-C
LB dataset																
Model 1 (Log. Reg.)	0.259	84.8			0.95 (0.90–0.99)						1.72 (1.16–2.91)					
Model 2 (Log. Reg., dicho)	0.203	88.2			0.95 (0.90–0.99)							4.43 (1.30–17.30)				
TB dataset																
Model 1 (AIC)	0.412	81.3		27.23 (2.06–659.20)		0.01 (0.00–0.18)				0.99 (0.99–1.00)		5.21 (1.17–29.20)		0.90 (0.81–0.99)		
Model 2 (Bootstrap)	0.242	85.2			0.91 (0.85–0.96)			1.05 (1.01–1.09)								
Model 3 (Lasso)	0.226	82.2										4.59 (1.48–15.96)				
Model 4 (Random forest^$^)			2		1		7	3	9	6			4	5	8	10

sqrt = square root; R^2^ = Coefficient of determination; R2 adj = adjusted coefficient of determination; $=first 10 most important variables ranked according to their importance; AIC = Akaike Information Criterion; SPADI = Shoulder Pain and Disability Index; SPADI-*P* = SPADI Pain subscale; SPADI-F = SPADI Function subscale; SPADI-C = SPADI change score at 5 weeks; SPADI-PC = SPADI Pain change sore at 5 weeks; SPADI-FC = SPADI Function change sore at 5 weeks; DOC = Duration of complaints; PET = Patient expectations of treatment; FABQ-PA = Fear avoidance beliefs questionnaire physical activity subscale; PCS = Pain catastrophizing scale; ANCOVA = Analysis of covariance; LB dataset = dataset based on the literature; TB dataset = dataset from the original trial.

## Discussion

The aim of this study was to explore factors predicting a successful or poor outcome after physiotherapy for SPS. The methodological approach was based on the assumption that analyzing data with different statistical approaches would increase the validity of the results. Analyses of the LB dataset identified patient expectancies as the most important predictor for both outcomes. For the TB dataset, FABQ-PA was a significant predictor in all models, and PET and SPADI-C were significant predictors in three out of four models for SPADI-1Y, with PET showing higher Beta-values. SPADI-P, SPADI-C, PET≥9, DOC, and PCS were identified in two out of four models as significant predictors of PGIC-1Y.

### Baseline differences between responders and nonresponders

Baseline differences could be identified for the SPADI, SPADI-P, SPADI-F, FABQ-PA, PET, and RER. All these factors except the RER played a more or less important role as predictors in the calculated models for both outcomes. However, other variables, such as duration of complaints or SPADI change scores, did not show any baseline differences between groups but were still identified as predictors in the calculated models. This emphasizes the fact that prediction cannot simply be based on those baseline variables, which obviously differ from either reference values or the central tendency of the sample.

### LB-dataset

The aim of applying results from the literature to our sample was to explore how well existing evidence could be replicated in our sample of SPS patients. Our results for the SPADI-1Y are in line with findings from Chester et al. [19], who also found that higher expectations, lower baseline pain and disability, and lower duration of complaints were significant positive predictors for the SPADI after six months in patients with shoulder pain. Additionally, Struyf et al. [18] identified a longer duration of complaints and greater baseline severity as strong negative predictors in patients with nontraumatic shoulder pain.

For the PGIC-1Y, higher expectations and lower SPADI pain scores were positive predictors and increased the chance for a positive outcome. This difference in identified predictors for each of our outcomes may be because the SPADI and the PGIC are different constructs and therefore have little intercorrelation [42]. However, other included predictors taken from the LB dataset, such as age or restriction of shoulder range of motion, were not significant in any of our models. This may reflect the difference between patients with SPS and the much broader group of patients with shoulder pain, differences in follow-up periods and measurement instruments, or the impossibility of simulating the full model with the available data from the original trial.

### TB dataset

Based on the comprehensive data used as putative predictors, we expected more variables to be relevant for prediction than those identified from the LB dataset. To verify and thus increase the validity of the results, different statistical models were calculated for each outcome. As significant positive predictors for the SPADI-1Y, lower FABQ-PA scores were identified in four out of four models, and higher expectations and greater SPADI change scores were identified in three out of four models. As seen in the LB dataset, expectations showed the strongest Beta-values. A SPADI pain score >46 at baseline was confirmed to be a negative predictor in two out of four models. According to the variables identified in at least two models for the PGIC-1Y, higher SPADI pain sores, longer duration of complaints, and higher pain catastrophizing scores were negative predictors, and a greater SPADI pain change score and expectations ≥9/10 were positive predictors. However, lower SPADI baseline scores and expectations ≥9/10 were also relevant as positive predictors in the much smaller model 2 from the LB dataset, which would support their relevance for prediction.

These results are partly in line with data from other studies showing long-term results in patients with SPS [20] or shoulder pain [43]. Although these authors included other factors in their analyses and thus ended up with different models, baseline pain and disability were consistently identified as significant negative predictors. However, these studies did not include psychological factors, did not find a significant association between psychological factors and outcomes, or found a dominance of clinical variables over psychological factors. In contrast, psychological factors such as expectations and fear avoidance beliefs were at least as relevant as clinical baseline or change sores in our analyses, which may again be explained by the specific subgroup of SPS patients.

### Identified predictors

#### Fear avoidance beliefs questionnaire – physical activity subscale

Fear avoidance beliefs were the most constant predictor for the SPADI-1Y in all the calculated models in the TB-dataset. This was surprising for two reasons: Firstly, because fear avoidance beliefs were not a relevant predictor of improvement in function at three months in this sample [21]. Secondly, because fear avoidance beliefs had not been identified in either the two systematic reviews [17, 18] or the cohort study [19]. This finding suggests that there may be differences between all patients with shoulder pain and patients with SPS in terms of their prognosis; it also indicates that fear avoidance beliefs should be considered in future analyses.

### Patients’ expectations of outcomes

In addition to fear avoidance beliefs, we identified expectations as the most consistent and strongest predictor in both the LB dataset and the TB dataset for both outcomes. The influence of patient expectancies on treatment outcomes in shoulder pain patients has also been investigated in other studies. For example, O’Malley et al. [44] reported a significant contribution of outcome expectancies to the prediction of changes in shoulder function. Patients with high expectations showed a clinically important improvement compared to those with low expectations. McDevitt et al. [45] reported that expectations of moderate relief were significantly associated with an improvement in global rating of change (GROC) ratings at 6 months in patients with chronic shoulder pain. In another study by Kvalvaag et al. [46], negative outcome expectations were the strongest negative predictor for the SPADI at the one-year follow-up. The results of these studies support our findings that expectations are a considerable predictor of outcomes in patients with SPS. The fact that positive expectations were consistently associated with better outcomes in other body regions and health problems [47, 48] also supports the importance of expectations as a so-called “contextual” predictor for outcomes. Furthermore, patient expectations are an important confounding aspect in clinical trials, and their assessment may therefore help to better interpret therapeutic outcomes from clinical trials [49].

### Greater disability and pain at baseline

These factors are among the most often identified predictors of negative short- and long-term outcomes in patients with shoulder pain [17–19, 43]. While our results confirm the importance of these two baseline variables as predictors for outcome, we found a different predictive relevance of the SPADI and its subscores for the SPADI-1Y and the PGIC-1Y, although we expected these variables to have similar importance for both outcomes. These differences might be because these outcomes may represent different constructs [42] and because PGIC ratings are more influenced by current status [50] than the SPADI is. Although baseline pain and disability variables could be identified as significant predictors for both outcomes, psychological predictors, in our case fear avoidance beliefs and expectations, were at least equally important predictors.

### SPADI change scores

We included short-term change scores of the SPADI, its subscales for pain and disability, and average pain intensity to analyze their possible contribution to prognosis This was done for two reasons. Firstly, against the background that, from a clinical perspective, an initial improvement in symptoms, among other things, seems to be a promising indicator for a potentially positive development and is thus relevant for prognosis. Secondly, there is evidence in the literature that short-term changes in combination with baseline data can contribute to an improved prognosis in patients with low back or shoulder pain [30]. However, the evidence is currently insufficient and simply does not exist for patients with SPS.

SPADI change scores were significant predictors of SPADI-1Y scores in all models. For PGIC-1Y, only two models included the SPADI change score as a predictor. However, these results deliver a first impression about the relevance of SPADI change scores in patients with SPS. A course over a 5-week period seems to be a promising indicator for further development and progression and should therefore be considered in future prediction research.

### Duration of complaints

The duration of complaints is frequently mentioned in the literature as a negative predictor of short- and long-term shoulder pain, often in combination with high disability or pain scores [17, 18, 43]. It was also a significant predictor in some of our analyses for both outcomes, but with low Beta-values. Interestingly, duration of complaints was a significant predictor in this sample of improvement in function at the three-month follow-up [21], but as observed for fear avoidance beliefs, the importance of putative predictive variables may change over the course of a rehabilitation process.

Other putative predictors for outcome mentioned in the literature showed no correlation with outcomes in our sample. For example, Braun et al. [51] identified ten predictive factors based on the literature and expert consensus and analyzed their contribution to the Western Ontario Rotator Cuff Index (WORC) at three months in a German sample with rotator cuff disorders. Among these factors, only pain catastrophizing significantly contributed to patient outcomes in their analyses. We analyzed the same or similar data for eight of these factors (age, sex, physical demands, disability, pain, symptom duration, and pain catastrophizing). Of these, we found a prognostic relevance for disability, pain, symptom duration, and pain catastrophizing for both outcomes, with catastrophizing being the weakest predictive factor for SPADI-1Y, which was apparent in only one model. The differences between the results of Braun et al. [51] and our results may be due to the different endpoints, different time points chosen for analysis, or the different definitions of eligibility criteria.

Other reasons why we could not find a contribution of other positive or negative factors mentioned in the literature to our outcome variables may exist. First, the contribution of independent variables to a predictive model might be linked to characteristics not included or unknown. Second, the results may be to some degree dependent on the definition of the targeted complaint, even if some of the identified predictors seem to be generic for musculoskeletal disorders in general [16, 52]. Third, different models could lead to different conclusions about the importance of the included factors. Fourth, the importance of baseline factors regarding their contribution to outcomes may change over time [21].

### Methodological considerations

We used the rating “much better” on the PGIC scale as a cut-off value to divide the sample into responders and nonresponders. This was to increase the likelihood that the rating given would actually reflect a significant change for the patient [31]. Nevertheless, this decision is subjective to a certain extent. The use of PGIC to divide a sample into responders and nonresponders must also be discussed against the background of the existing scientific literature. There are both results that tend to recommend the use of a patients’ overall impression about improvement [53, 54], and results that argue against it [50]. In our view, the assessment of what constitutes a truly significant change in overall symptoms for the individual is essential information that can only partially be captured with a measurement instrument such as the SPADI. However, this approach can be seen as a limitation of this work. Although the initial selection of putative predictors for the LB-dataset was based on sound literature, the actual LB dataset was limited for several reasons. First, we could only include those factors for which we had corresponding data from the original trial. Consequently, other factors could not be considered. Second, due to our sample size, we could include only a certain number of variables in the analyses, which was also reflected in the limited selection of variables from the bootstrapping and the random forest regression. Third, yet importantly, this was a secondary analysis, and no power calculations were performed. Therefore, the sample was possibly too small for the contributions of some of these variables to become significant. Furthermore, dichotomizing independent variables leads to a certain loss of information.

To guarantee good external validity for the results of the original trial, the population was defined based on clinical examination results, and eligibility criteria were chosen accordingly. However, they were defined with the intention of selecting participants for a randomized controlled trial. Although patients may vary to a certain degree in some of the baseline variables, e.g., duration of complaints, the possible homogeneity of our sample regarding their complaints due to these eligibility criteria may also have influenced our results. Furthermore, expectations were assessed with only one question of the CEQ, and fear avoidance beliefs were assessed with an adapted version of the FABQ; therefore, the measurement validity of these variables can be questioned.

### Clinical implications

If expectations are a relevant prognostic factor for outcome in physiotherapy, it would be relevant to know how expectations can be positively influenced in a clinical setting to increase the chance for a good treatment result. A possible starting point could be the contextual model [55] and strategies derived from it, such as (i) building a trusting therapeutic bond that may induce positive expectations toward physiotherapy and the therapist but also facilitating shared decision-making regarding goals and therapy means to achieve these goals, (ii) providing a logical and acceptable explanation of the underlying problem or pathology and a potentially effective treatment that is in line with the preceding explanations, (iii) providing sufficient information to patients to improve their understanding of therapy, its positive effects, and possible side effects, and (iv) activating personal resources that may support individual coping abilities.

However, scientific evidence for such strategies in a physiotherapy setting is scarce, and thus, more research is needed about the influence of expectations and their impact on outcomes. This is also closely linked to the question of whether specific interventions to increase expectations should precede SPS-specific therapy if low expectancy scores are present. Depending on the results, physiotherapists need to be trained to effectively identify and address patients with low expectancy scores to increase the chance for an effective treatment outcome or at least prevent them from ineffective interventions.

## Conclusions

Higher expectations and SPADI change scores were positive predictors, and higher fear avoidance beliefs and higher baseline disability and pain levels were negative predictors of long-term outcomes in patients with SPS. Therefore, these factors should be considered by therapists during history taking and physical examination to adapt treatment accordingly and thus to optimize results. In particular patients’ expectations, the predictor most frequently identified as significant in our models, as well as fear avoidance beliefs and SPADI short-term change scores, two variables found to be relevant predictors in this patient group should be addressed in further prediction studies. The SPADI itself should be used as a baseline assessment and as an instrument to assess short-term development as a predictor in clinical practice and further studies.

Due to its explorative nature and the limitations mentioned above, hypotheses and recommendations generated from our results need to be reviewed in further studies.

### Electronic supplementary material

Below is the link to the electronic supplementary material.

Supplementary Material 1

Supplementary Material 2

Supplementary Material 3

Supplementary Material 4

Supplementary Material 5

Supplementary Material 6

Supplementary Material 7

Supplementary Material 8

Supplementary Material 9

Supplementary Material 10

## Data Availability

The data that support the findings of this study are not openly available due to reasons of sensitivity and are available from the corresponding author upon reasonable request. Data are located in a controlled access data repository at https://zenodo.org.
