# Supplementary material for: Factors predicting long-term outcomes following physiotherapy in patients with subacromial pain syndrome: a secondary analysis
Source: BMC Musculoskelet Disord. 2024 Jul 24;25:579. doi: 10.1186/s12891-024-07686-6 (PMC11267964; doi:10.1186/s12891-024-07686-6)
Supplement: Supplementary file 1 — Supplementary Material 1 [file 12891_2024_7686_MOESM1_ESM.pdf]

Additional file 01:

Additional Figure 1: LB dataset, PGIC-1Y, ROC curve model 1

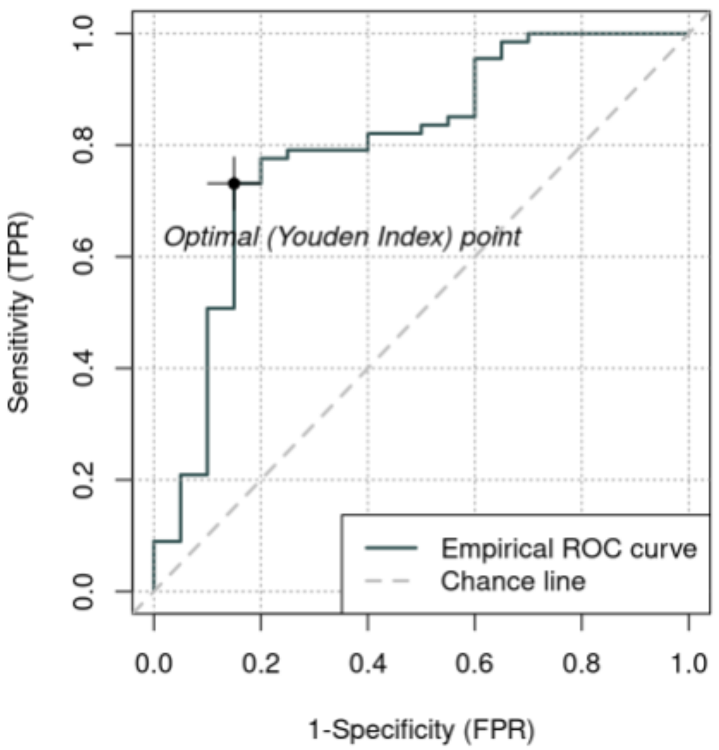

(TPR = true positive rate, FPR = false positive rate)

Additional Figure 2: LB dataset, PGIC-1Y, ROC curve model 2

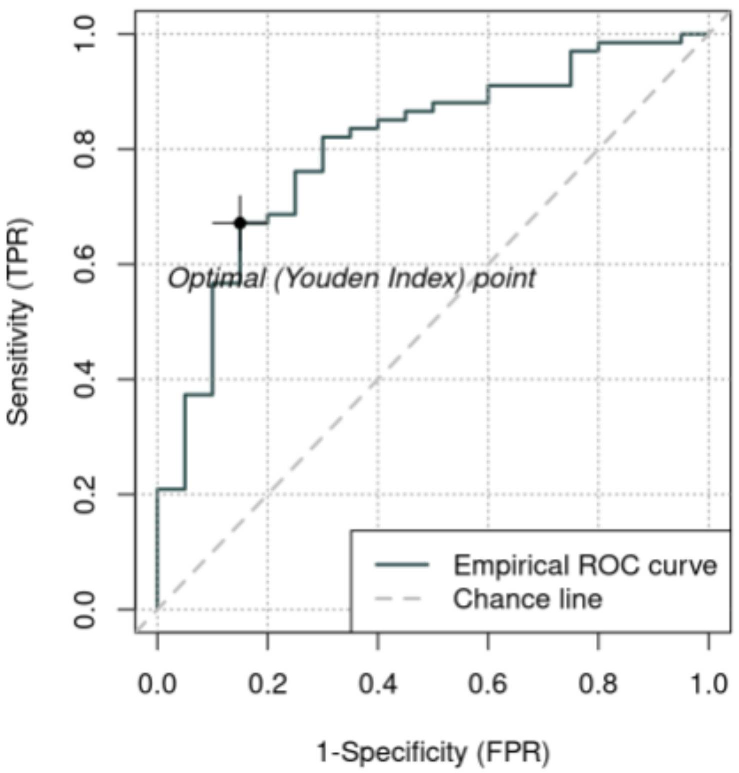

(TPR = true positive rate, FPR = false positive rate)
