# Supplementary material for: Factors predicting long-term outcomes following physiotherapy in patients with subacromial pain syndrome: a secondary analysis
Source: BMC Musculoskelet Disord. 2024 Jul 24;25:579. doi: 10.1186/s12891-024-07686-6 (PMC11267964; doi:10.1186/s12891-024-07686-6)
Supplement: Supplementary file 2 — Supplementary Material 2 [file 12891_2024_7686_MOESM2_ESM.pdf]

Additional file 02:

Additional Table 1. TB dataset, SPADI-1Y.sqrt, model 1: Coefficients (stepwise model selection through AIC)

Predictors	Estimates	CI	p
(Intercept)	1.53	0.10 – 2.97	0.037*
DOC	0.00	0.00 – 0.01	0.049*
SPADI-P > 46	1.54	0.62 – 2.46	0.001**
FABQ-PA	0.09	0.00 – 0.17	0.040*
PET ≥ 9	-1.18	-2.03 – -0.32	0.007**
SPADI-C	-0.09	-0.14 – -0.03	0.003**
SPADI-FC	0.07	0.01 – 0.12	0.016*
Observations:	87		
R^2 / R^2 adj:	0.396 / 0.351	AIC: 363.8	

CI= Confidence Interval; p=p-value; *=p<0.05; **=p<0.01; ***=p<0.001; R^2=Coefficient of determination; R^2 adj.=Adjusted coefficient of determination; AIC=Akaike information criterion; sqrt=square root
