# Supplementary figures and images for: Factors predicting long-term outcomes following physiotherapy in patients with subacromial pain syndrome: a secondary analysis
Source: BMC Musculoskelet Disord. 2024 Jul 24;25:579. doi: 10.1186/s12891-024-07686-6 (PMC11267964; doi:10.1186/s12891-024-07686-6)

Additional file 03:

Additional Figure 3: TB dataset, SPADI-1Y, bootstrapping results

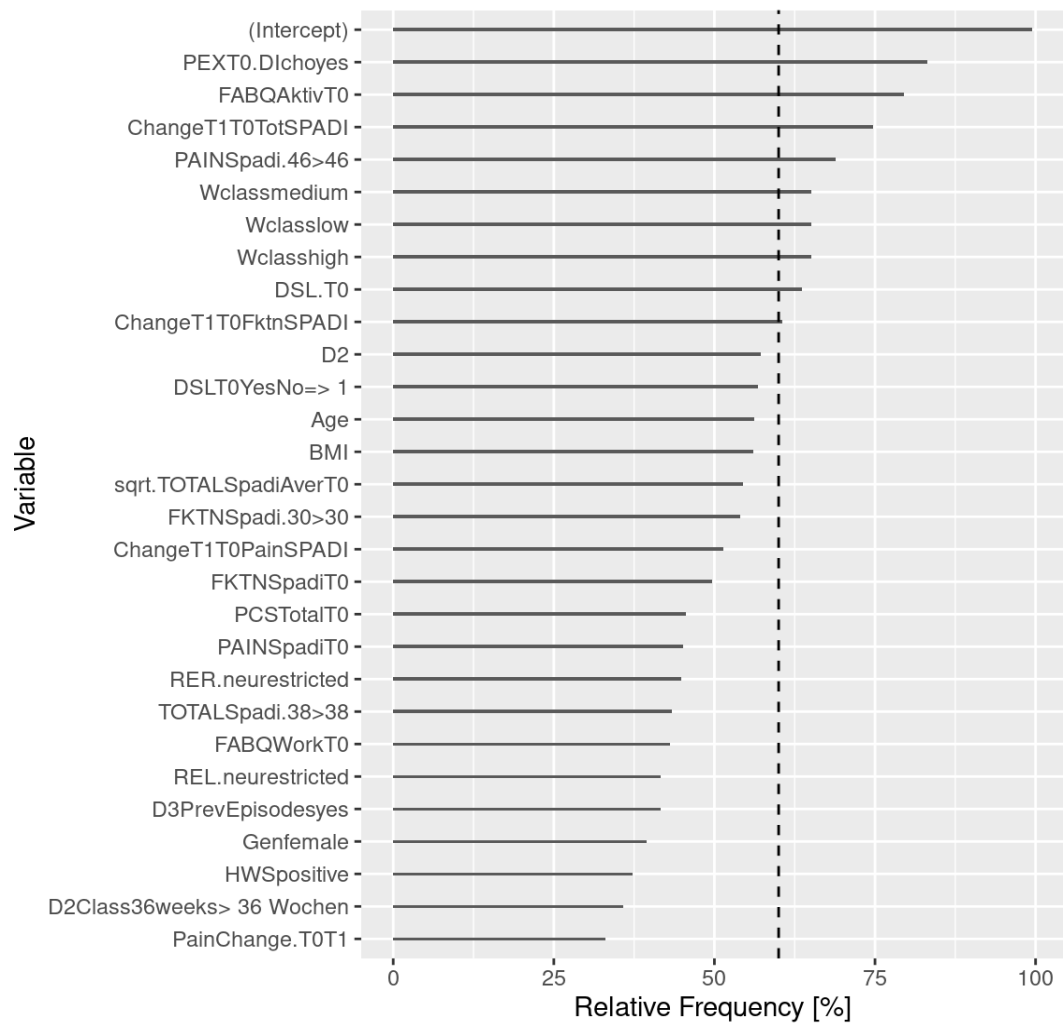

Supplement: Supplementary file 3 — Supplementary Material 3 [file 12891_2024_7686_MOESM3_ESM.pdf]

Additional file 04:

Additional Figure 4: TB dataset, SPADI-1Y, model 4, random forest

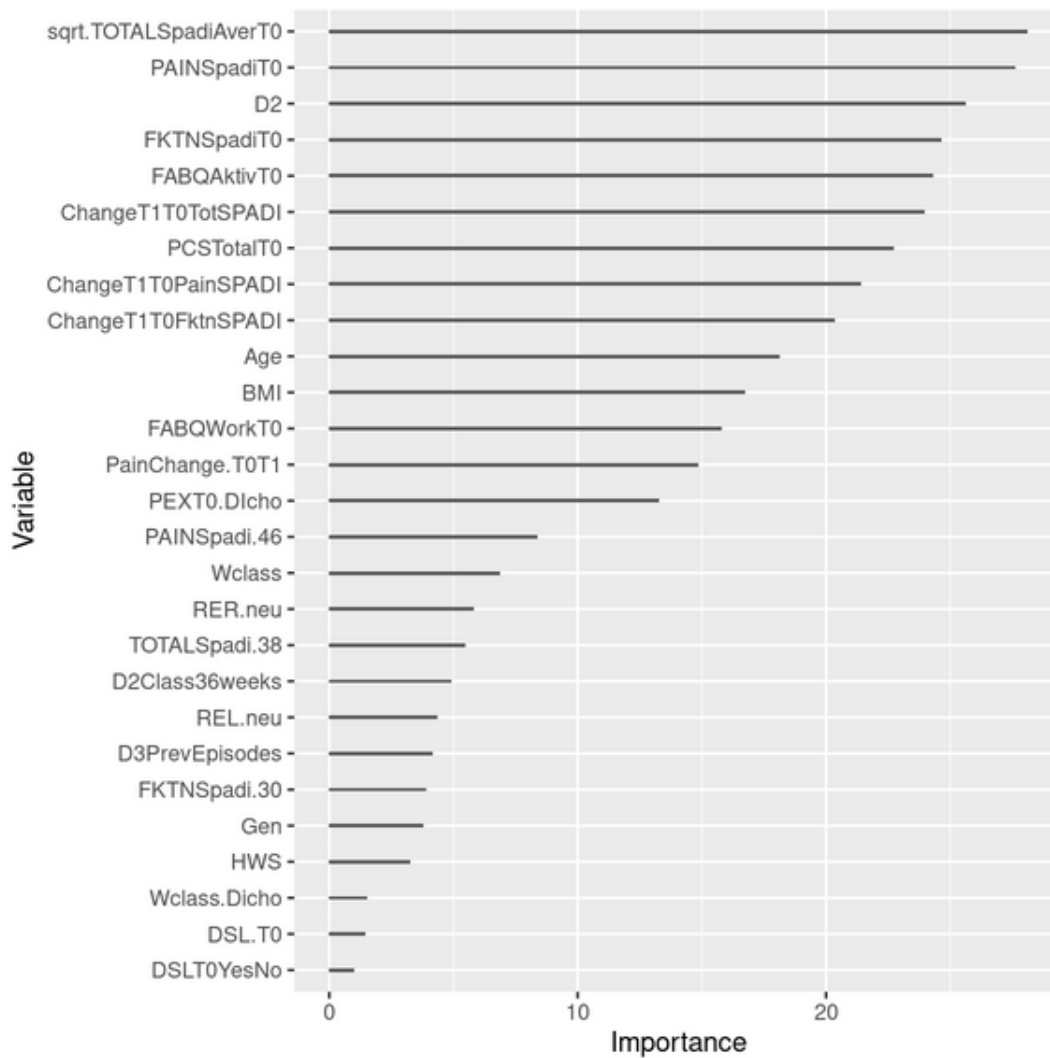

Supplement: Supplementary file 4 — Supplementary Material 4 [file 12891_2024_7686_MOESM4_ESM.pdf]

Additional file 06:

Additional Figure 5: TB dataset, PGIC-1Y, bootstrapping results

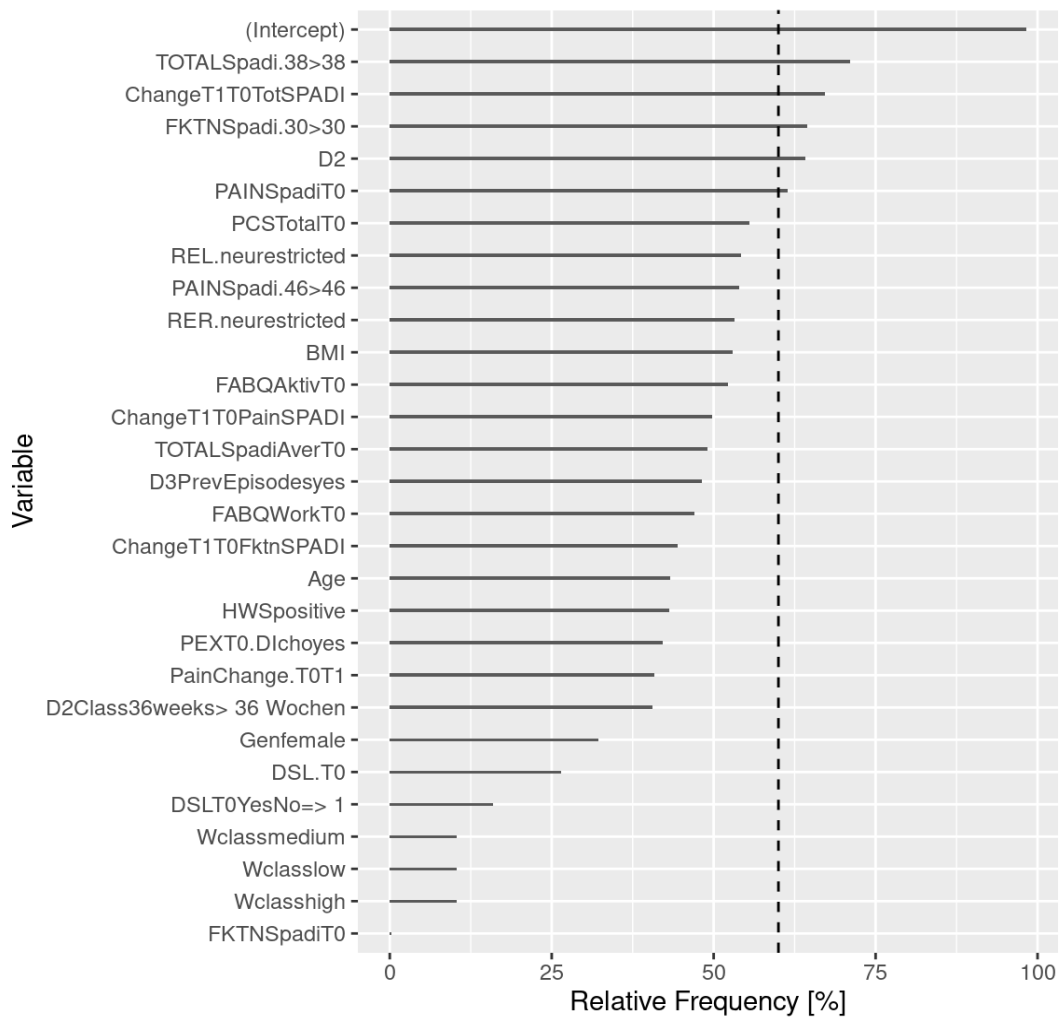

Supplement: Supplementary file 6 — Supplementary Material 6 [file 12891_2024_7686_MOESM6_ESM.pdf]

Additional file 09:

Additional Figure 9: TB dataset, PGIC-1Y, random forest

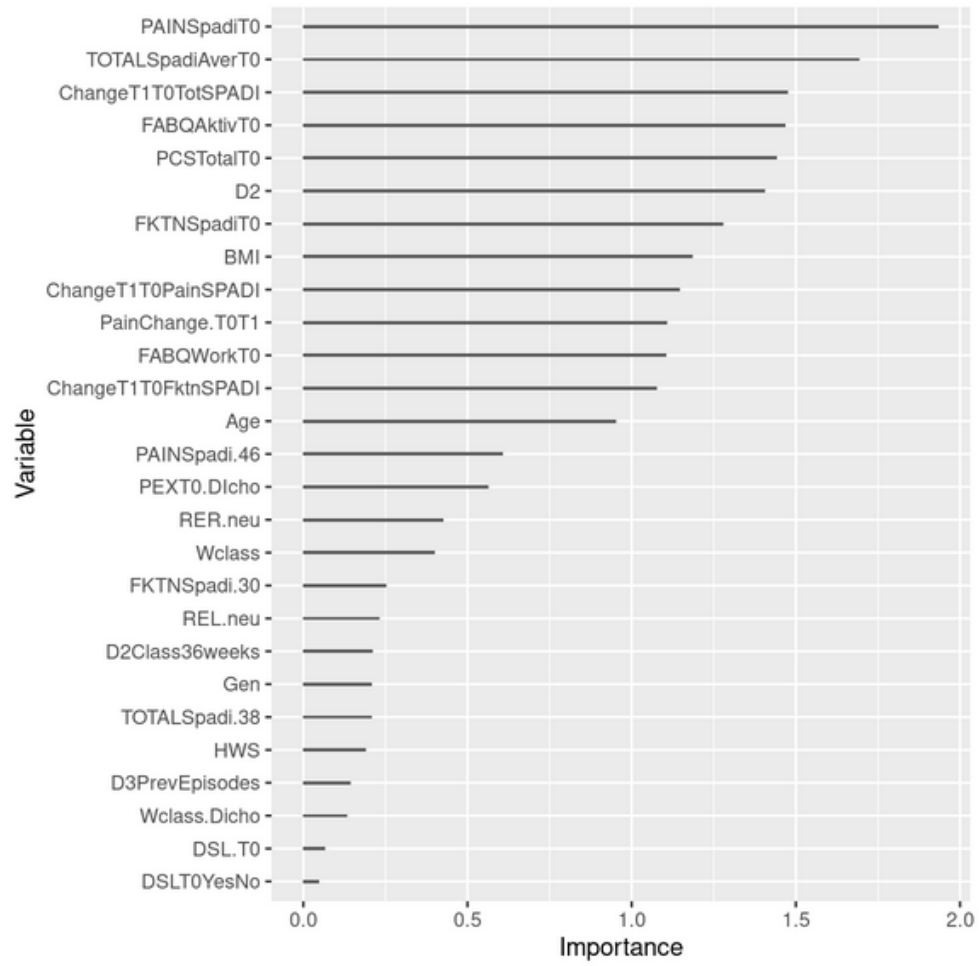

Supplement: Supplementary file 9 — Supplementary Material 9 [file 12891_2024_7686_MOESM9_ESM.pdf]
