# Supplementary material for: Factors predicting long-term outcomes following physiotherapy in patients with subacromial pain syndrome: a secondary analysis
Source: BMC Musculoskelet Disord. 2024 Jul 24;25:579. doi: 10.1186/s12891-024-07686-6 (PMC11267964; doi:10.1186/s12891-024-07686-6)
Supplement: Supplementary file 5 — Supplementary Material 5 [file 12891_2024_7686_MOESM5_ESM.pdf]

Additional file 05:

Additional Table 2. TB dataset, PGIC-1Y, model 1: Coefficients (stepwise model selection through AIC)

	Responder		
Predictors	Odds Ratios	CI	p
(Intercept)	0.11	0.00 – 10.63	0.362
BMI	1.21	1.01 – 1.49	0.055
DOC	0.99	0.99 – 1.00	0.043*
PE	6.91	0.93 – 83.38	0.084
SPADI > 38	27.23	2.06 – 659.20	0.023*
SPADI-P > 46	0.01	0.00 – 0.18	0.003**
PCS	0.90	0.81 – 0.99	0.038*
PET ≥ 9	5.21	1.17 – 29.20	0.039*
REL	0.21	0.03 – 1.20	0.101
SPADI-C	1.26	1.05 – NA	0.067
SPADI-PC	0.93	NA – 1.00	0.205
SPADI-FC	0.87	0.64 – 1.00	0.090
Observations:	87		
R^2 Tjur: 0.412	AIC: 81.32		

CI= Confidence Interval; p=p-value; *=p<0.05; **=p<0.01; ***=p<0.001; R^2 Tjur=Coefficient of determination; AIC=Akaike information criterion
