# Supplementary material for: Factors predicting long-term outcomes following physiotherapy in patients with subacromial pain syndrome: a secondary analysis
Source: BMC Musculoskelet Disord. 2024 Jul 24;25:579. doi: 10.1186/s12891-024-07686-6 (PMC11267964; doi:10.1186/s12891-024-07686-6)
Supplement: Supplementary file 8 — Supplementary Material 8 [file 12891_2024_7686_MOESM8_ESM.pdf]

Additional file 08:

Additional Table 3. TB dataset, PGIC-1Y, model 3: Coefficients (Lasso)

	Responder		
Predictors	Odds Ratios	CI	p
(Intercept)	9.18	1.13 – 83.36	0.041*
PET ≥ 9	4.59	1.48 – 15.96	0.011*
SPADI-P	0.99	0.94 – 1.04	0.564
SPADI-P >46	0.21	0.03 – 1.39	0.111
Observations:	87		
R^2 Tjur: 0.226	AIC: 82.24		

CI= Confidence Interval; p=p-value; *=p<0.05; **=p<0.01; ***=p<0.001; R^2 Tjur=Coefficient of determination; AIC=Akaike information criterion

Additional Figure 8: TB dataset, PGIC-1Y, ROC curve Lasso

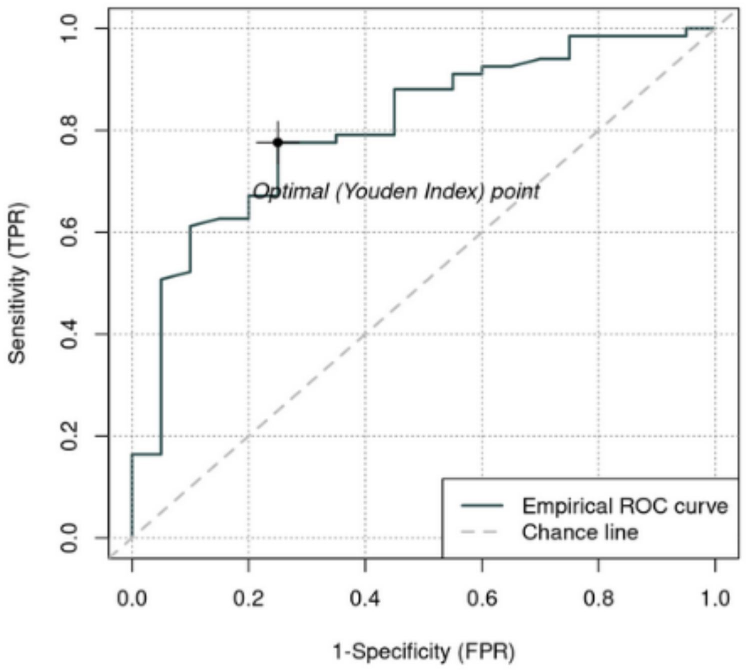

(TPR = true positive rate, FPR = false positive rate)
