# Supplementary material for: Factors predicting long-term outcomes following physiotherapy in patients with subacromial pain syndrome: a secondary analysis
Source: BMC Musculoskelet Disord. 2024 Jul 24;25:579. doi: 10.1186/s12891-024-07686-6 (PMC11267964; doi:10.1186/s12891-024-07686-6)
Supplement: Supplementary file 10 — Supplementary Material 10 [file 12891_2024_7686_MOESM10_ESM.pdf]

Factors predicting long-term outcomes following physiotherapy in patients with subacromial pain syndrome: A secondary analysis

Thilo O. Kromer, Matthias Kohl, Caroline H.G. Bastiaenen

List of Additional files

Additional_file01.pdf:	Additional Figure 1: LB-dataset, PGIC-1Y, ROC curve model 1. Additional Figure 2: LB-dataset, PGIC-1Y, ROC curve model 2
Additional_file02.pdf:	Additional Table 1. TB-dataset, SPADI-1Y.sqrt, model 1: Coefficients (stepwise model selection through AIC)
Additional_file03.pdf:	Additional Figure 3: TB-dataset, SPADI-1Y, bootstrapping results
Additional_file04.pdf:	Additional Figure 4: TB-dataset, SPADI-1Y, model 4, random forest
Additional_file05.pdf:	Additional Table 2. TB-dataset, PGIC-1Y, model 1: Coefficients (stepwise model selection through AIC)
Additional_file07.pdf:	Additional Figure 5: TB-dataset, PGIC-1Y, bootstrapping results
Additional_file07.pdf:	Additional Figure 6: TB-dataset, PGIC-1Y, ROC model 1. Additional Figure 7: TB-dataset, PGIC-1Y, ROC model 2
Additional_file08.pdf:	Additional Table 3. TB-dataset, PGIC-1Y, model 3: Coefficients (Lasso); Additional Figure 8: TB-dataset, PGIC-1Y, ROC Lasso
Additional_file09.pdf:	Additional Figure 9: TB-dataset, PGIC-1Y, random forest
